# Development and validation of a video-assisted liver biopsy technique using a minimally-invasive device

**DOI:** 10.1186/s12876-023-02740-4

**Published:** 2023-04-11

**Authors:** Alexandra Mano Almeida, Hermano Alexandre Lima Rocha, David Augusto Batista Sá Araújo, Paulo Goberlânio de Barros Silva, Luís Pires de Melo Filho, Gleydson César de Oliveira Borges

**Affiliations:** 1 Unichristus University Center. Fortaleza, Fortaleza, CE Brazil; 2 Department of Global Health and Population, Harvard T. H. Chan School of Public Health, Boston, MA USA; 3 Department of Community Health, Federal University of Ceará, Fortaleza, CE Brazil

**Keywords:** Biopsy, Needle, Biopsy, Large-Core Needle, Laparoscopes, Hemorrhage, Liver Cirrhosis

## Abstract

**Background:**

Percutaneous liver biopsy is the gold standard technique for establishing the cause of cirrhosis and liver disease activity assessment. However, some cases of steatohepatitis or other chronic liver diseases show a high number of false negative results in samples obtained via the percutaneous route. This fact justifies performing a liver biopsy via the laparoscopic route. However, this is an expensive technique, with morbidities associated with pneumoperitoneum and anesthetic complications. The main objective of this study is to develop a video-assisted technique that uses only a minimally-invasive device for the liver biopsy and the optical trocar. Without additional trocars, this technique constitutes a less invasive procedure than the existing techniques in clinical practice.

**Methods:**

This is a device development and validation study and patients submitted to abdominal laparoscopic surgery and required liver biopsy for moderate to severe steatosis were recruited. The patients were randomized into two groups: laparoscopic liver biopsy technique (*n* = 10, control group) and mini-laparoscopic liver biopsy technique (*n* = 8, experimental group). The times associated with procedure performance in both groups were evaluated using the Mann–Whitney or Kruskal–Wallis tests according to data distribution.

**Results:**

At baseline, there was no statistical difference regarding gender and type of surgery. The experimental group had a significantly shorter time compared with the group that underwent the traditional procedure in mean procedure time (*p* = 0.003), biopsy time (*p* = 0.002) and hemostasis time (*p* = 0.003).

**Conclusions:**

The mini-laparoscopic biopsy device and technique showed to be capable of safely obtaining sufficient tissue samples, which was minimally invasive and in a shorter time than the classic technique.

## Background

Liver disease is one of the most significant health problems in the United States. According to the Centers for Disease Control and Prevention (CDC), cirrhosis and other chronic liver diseases are considered the 12^th^ leading cause of death in the United States and are responsible for more than 60,000 deaths annually [1, 2]. They constitute the sixth leading cause of death between the ages of 35 and 55 in North-American individuals [3–5]. Approximately 30,000 new patients are diagnosed with cirrhosis at tertiary health centers each year. However, only 10 to 15% correspond to alcoholic cirrhosis [2, 6].

The liver parenchyma is organized into microscopic functional units called lobules. A hepatic lobule is described as a functional polyhedral unit, in which each angle houses the so-called portal triad (the hepatic artery branch, the hepatic portal vein branch, and the biliary ducts [4, 5]. Each lobule houses a central hepatic venule and the entire space between this central vein and the portal triad is filled with the basal cell of the hepatic parenchyma, i.e., the hepatocyte [4, 5]. Because of these anatomical and histological characteristics, until the mid-1800s, any procedure involving the liver was considered dangerous and often impossible. For decades, all surgical techniques were considered uncertain and risky [4]. It was observed that no matter how small the resected area, there was blood and bile drainage from the exposed surgical surface.

Due to this risk during resections and aiming at greater diagnostic accuracy, liver biopsies started being used for the diagnosis and monitoring of liver diseases. In 1958, Menghini planned and published an innovative method, called the “One-second needle biopsy of the liver”, of which performance consists in puncturing the liver parenchyma with a biopsy needle through a trans-costal access with the patient lying in the horizontal dorsal decubitus position [7]. In subsequent years, this technique was improved and modified due to the introduction of better needle biopsy devices [8].

Although the biopsy is a more favorable option for the diagnosis and monitoring of patients with liver disease, patients who need liver biopsy very often have liver function damage and abnormal coagulation status [9]. And that is considered a potential reason that leads to major bleeding [10]. The percutaneous liver biopsy (PLB) should not be performed when the patient is uncooperative, in cases of coagulopathy, in the presence of ascites and in morbidly obese patients. In these situations, a sample of liver tissue must be obtained through another approach, as these patients are at high risk of post-biopsy bleeding and, thus, liver biopsy using the laparoscopic or video-assisted technique is the chosen procedure. The laparoscopic biopsy allows an adequate tissue evaluation under direct view, with direct and immediate bleeding control [11]. The need to be performed by a trained team and under general anesthesia has limited its use for several decades [12].

However, this procedure is not free from complications, with the main ones being intraperitoneal or abdominal wall trauma and bleeding [11, 12]. Thus, to evaluate the use of a minimally-invasive device to perform biopsies in these situations, we aimed to develop a new method that allows video-assisted liver puncture for biopsy, which constitutes a less invasive technique than the existing ones in clinical practice.

## Methods

### Study design and setting

A biopsy device development and validation study was carried out in two large tertiary surgical hospitals in the city of Fortaleza, state of Ceará, Brazil.

### Sample

Based on the study by Eisemberg et al. (2003), which observed that 83% of patients had painful symptoms after liver biopsies 30 min after the surgical procedure *versus* 39% after 24 h [13], we estimated that it was necessary to evaluate a total of 24 patients, equally divided into the two groups, aiming to obtain a sample that had 80% of power and 95% confidence (Fleiss method with continuity correction) and was a representative sample of liver biopsies.

Twenty-four (24) patients who were about to undergo abdominal laparoscopic surgery and who had an ultrasound finding of moderate to severe hepatic steatosis were then recruited to participate in the study. These patients had access to hospitals belonging to the research environment.

*Inclusion criteria*

- Patients with moderate to severe hepatic steatosis, submitted to abdominal laparoscopic surgery for another underlying disease;
- Patients who needed a liver biopsy for etiological confirmation or staging of disease activity.

*Exclusion Criteria*

- Refusal to participate in the study. 
  ••Patients diagnosed with hepatocellular carcinoma (HCC).
- The following risk factors:
  - Patients with INR > 1.8 and/or platelets < 70,000/mm.^3^
  - Patients with coagulopathy

### Research protocol

The device designed for the research consists of a needle shaft covered by insulating material (plastic, silicone or polyurethane). It was not necessary to manufacture the device, as a product with these characteristics is already available in the clinical practice of health services. It was only adapted for the purpose proposed in the study.

The needled catheter was used for the first time in 1945 aiming at venipuncture for long-term intravenous therapy [14, 15]. It was improved in 1957 and the needle was covered with a flexible silicone or polyurethane. The needle is removed at the time of catheter insertion, leaving only the plastic, siliconized or polyurethane catheter [14]. This constitutes the currently known venipuncture device. The Jelco® or Abocath® are used worldwide for the catheterization of peripheral veins. They are numbered and graded according to the gauge of the needle [15].

A number 14 Jelco® was used for the study, whose caliber of approximately 01 mm allows the passage of the 16G Trucut biopsy needle.

All procedures started with liver biopsy and subsequently, sequential surgery was performed. Thus, there was no influence of the previously scheduled surgery.

### Device systematization and validation

Twelve (12) patients were chosen to assess the device function. During the surgical procedure, the abdominal wall of the right hypochondrium was punctured in the midclavicular line, 1.0 cm below the right costal margin, forming a 90º angle with the skin. The puncture was performed with a number 14 Jelco®, its needle was removed and the silicone remained for the passage of the liver biopsy needle.

**Fig. 1:**
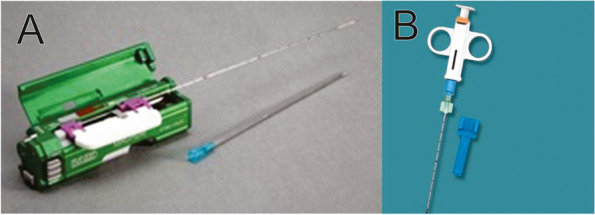
16G Trucut Needles. A Permanent base needle. B Disposable needle

Two standard types of biopsy needles were used to perform the procedure: the permanent base needle, in which only the metal that enters the parenchyma is replaced, with the trigger being reused after sterilization (Fig. 1A) and the needle is completely disposable, which consists of the metallic support and the trigger (Fig. 1B).

#### Study groups

The 24 patients included in the study were randomly allocated into one of the following groups, divided according to the liver puncture technique used for the biopsy. The patients in both groups were admitted to hospital and fasted for 8 h. The pre-anesthetic, intraoperative and post-anesthetic methods were performed according to the routine of the anesthesiologist, using previously established anesthetic procedures. The first procedure performed was the liver biopsy and then, the patient’s baseline surgery was performed.

#### Randomization

As this is an open study, all patients who agreed to participate in the study were aware of the biopsy technique used. The method used to obtain the liver parenchyma sample (whether conventional laparoscopic or mini-laparoscopy technique with the device) was defined through randomization, using a randomization list generated by the website http://www.randomization.com on August 20, 2019, with the patients being informed after the surgery. All procedures were performed by digestive system surgeons who had at least 6 years of training with experience in liver surgery and liver transplantation.

### “Laparoscopic Liver Biopsy Technique” – Control Group (*n* = 10)

**Fig. 2:**
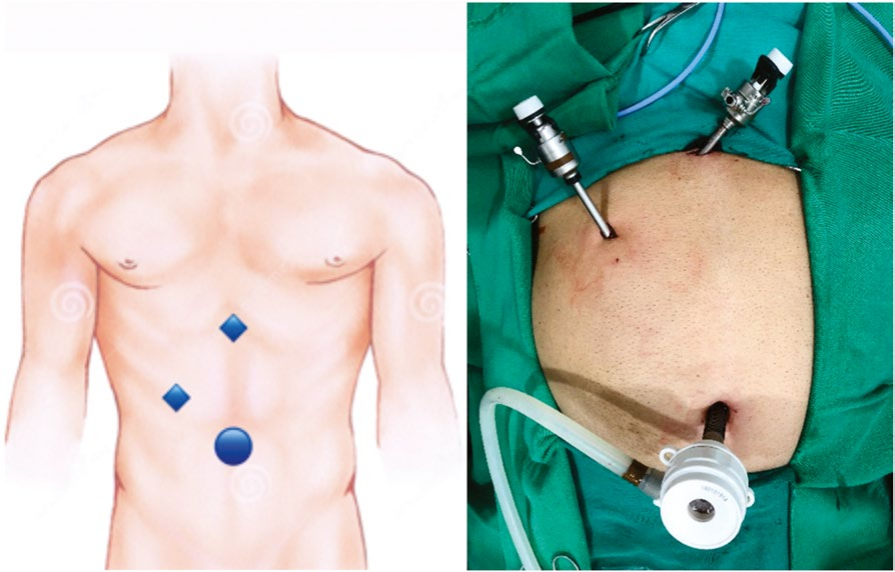
Trocar insertion sites in the laparoscopic technique. Sub-figure labelling: On the left the circle represents the 10 mm trocar of the optics and the diamonds represent the 05 mm trocars; on the right, the equivalent location in the surgical act

**Fig. 3:**
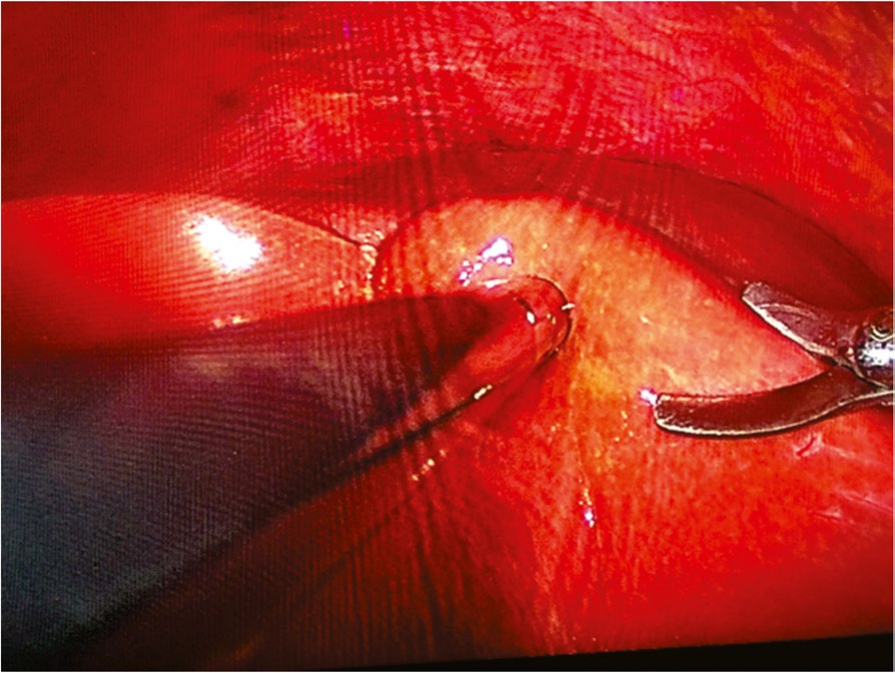
Biopsy of the liver parenchyma with scissors using the laparoscopic technique

The surgical technique consists of (Fig. 2):

1. Patient lying in the horizontal dorsal decubitus position under general anesthesia;
2. Asepsis, antisepsis and apposition of surgical drapes;
3. Supra-umbilical arcuate incision; dieresis by planes; opening of the aponeurosis and passage of the 10-mm trocar, followed by the creation of the pneumoperitoneum using the open technique;
4. 30° optical trocar apposition and cavity inventory;
5. Introduction of a 5-mm trocar in the epigastrium, below the xiphoid appendix and introduction of a 5-mm trocar, 1.0 cm below the right costal margin in the midclavicular line (both will be used for the surgeon’s movement);
6. The biopsy was performed with laparoscopic scissors (Fig. 3) and the exposed area is cauterized using a monopolar hemostatic forceps (Hook forceps);
7. Review of hemostasis and count of the material used during surgery.

### “Mini-Laparoscopy Liver Biopsy Technique”—Experimental group (*n* = 8)

**Fig. 4:**
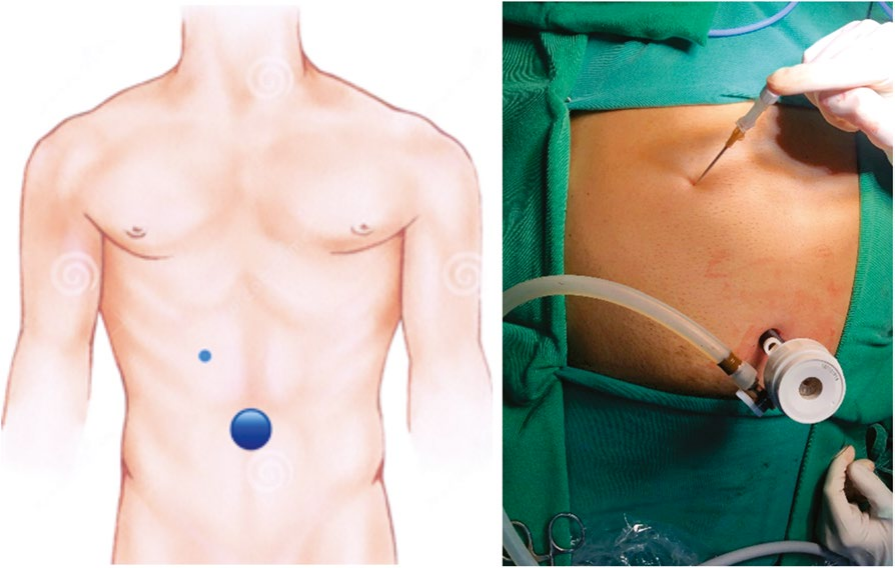
Trocar and device insertion sites in the mini-laparoscopy technique. Sub-figure labelling: On the left, schematically the larger circle represents the 10 mm trocar of the optics and the smaller circle represents the puncture point with the suggested device. On the right, follows the equivalent location in the surgical act

**Fig. 5:**
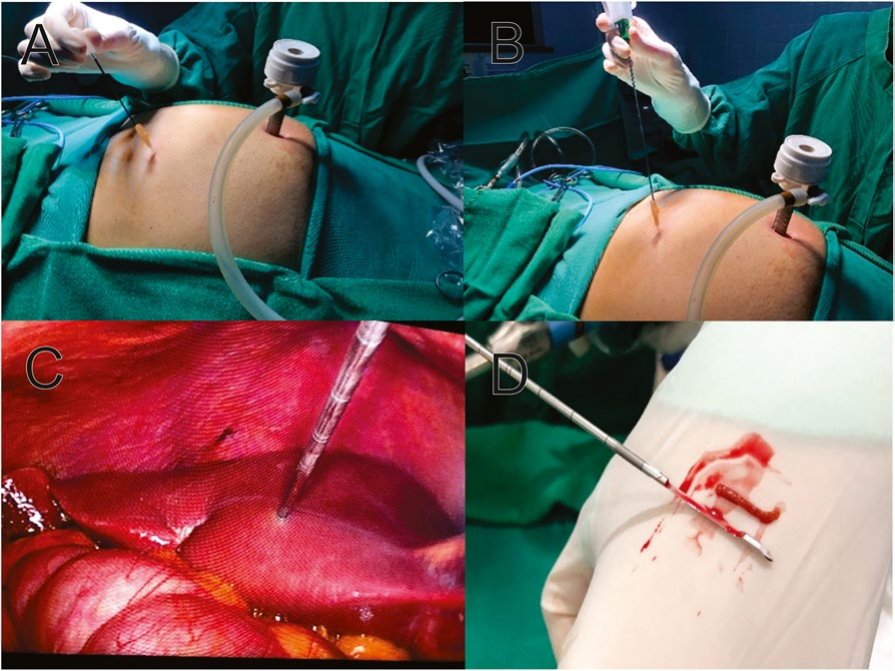
Steps performed to obtain the liver parenchyma biopsy specimen using a Trucut needle. A The needle is removed from the Jelco® and the silicone is maintained; (B) The Trucut needle is placed through the silicone strut; (C) Use of the Trucut needle to obtain the biopsy material; (D) Detail of the obtained fragment measuring 02 cm

The surgical technique consists of (Fig. 4):

1. Patient lying in the horizontal dorsal decubitus position under general anesthesia;
2. Asepsis, antisepsis and apposition of surgical drapes;
3. Supra-umbilical arcuate incision; dieresis by planes; opening of the aponeurosis and passage of the 10-mm trocar, followed by creation of pneumoperitoneum using the open technique;
4. 30° optical trocar apposition and cavity inventory;
5. Device insertion in the right hypochondrium, 1.0 cm below the right costal margin, in the midclavicular line, forming a 90º angle with the skin;
6. The needle is removed from the device and the silicone is maintained;
7. Placement of the cocked biopsy needle inside the device silicone strut, which is fired for the biopsy (Fig. 5);
8. The biopsy fragment is removed and the needle is reintroduced;
9. The needle is kept in contact with the biopsy site and tissue cauterization is performed by touching the monopolar electric scalpel to the device needle (there is no transmission to the skin, subcutaneous tissue or muscle, as the device has electrical insulation);
10. Review of hemostasis and count of the material used during surgery.

### Measures

- Total procedure time: considered from the beginning of the pneumoperitoneum until the end of the cauterization of the biopsied liver parenchyma. 
  - ◦ Time measured with a stopwatch and counted in minutes.

- Time of liver biopsy with placement of 0.5-mm trocars (laparoscopic technique) + laparoscopic scissors biopsy: considered from the placement of the 1^st^ trocar until removal of the biopsy sample.
  - ◦Time measured with a stopwatch and counted in minutes.

- Time of liver biopsy with device placement (mini-laparoscopy technique) + biopsy needle apposition: considered from device placement until biopsy sample removal.
  - ◦ Time measured with a stopwatch and counted in minutes.

- Representativeness of the sample:
  - ◦ Size of the biopsy sample measured in centimeters.

- Time of hemostasis: considered from the removal of the biopsy sample to the complete cauterization of the liver parenchyma. 
  - ◦ Time measured with a stopwatch and contact in seconds.

- Occurrence of hemorrhage: 
  - ◦Measured by the dichotomous criterion (YES/NO), taking into account the patient’s hemodynamic instability (tachycardia or hypotension). 
  - ◦ If positive for hemorrhage, assess resolution by the dichotomous criteria (YES/NO).

- Occurrence of bile leak:
  - ◦ Measured by the dichotomous criterion (YES/NO), taking into account the leak of bile from the biopsy area.
  - ◦ If positive for bile leak, assess the resolution using the dichotomous criterion (YES/NO).

- Quality of the biopsy sample:
  - ◦Assessed in the anatomopathological examination according to the number of individualized portal spaces (using the same laboratory).
  - ◦ ≥ 5 portal spaces: satisfactory sample.
  - ◦ < 5 portal spaces: unsatisfactory sample.

- Handling of the developed device: the surgeon's degree of satisfaction with the device position and design. The data were classified according to the response using the Likert scale [16].
- Ergonomics of the developed device: the surgeon's degree of satisfaction with the device's degree of comfort and safety. The data were classified according to the response using the Likert scale.
- Accessibility provided by the developed device: the surgeon's degree of satisfaction regarding access to the abdominal cavity and the performance of the biopsy. The data were classified according to the response using the Likert scale.

### Statistical analysis

Clinical data were expressed as absolute and percentage frequency and analyzed using Fisher's exact test or Pearson's chi-square test. Surgical times were expressed as mean and standard deviation, submitted to the Shapiro–Wilk normality test and analyzed using the Mann–Whitney or Kruskal–Wallis tests. The analyzes were performed using the SPSS software, version 20.0 for Windows, adopting a 95% confidence level.

### Ethics

All patients who agreed to participate in the study signed the Free and Informed Consent Form and were previously informed about the conditions and objectives of the study. All of them were free to withdraw from the study at any time, without entailing any kind of damage to their physical or emotional integrity. The National Research Ethics Commission of Brazil (CONEPE), in accordance with the attributions defined in CNS Resolution number 466 of 2012 and CNS Operational Standard number 001 of 2013, approved the research project under Opinion number 3.515.278 and CAAE—01152918.9.0000.5049. It was registered under trial number RBR-4n6rqyj in 02/02/2023.

## Results

Most patients were females, aged over 45 years, and had been previously submitted to video cholecystectomy and had an indication for liver biopsy due to severe hepatic steatosis. There was no significant difference regarding distribution by gender (*p* = 0.092), age (*p* = 1,000) and type of surgery (*p* = 0.104) between the study groups. There was a higher prevalence of patients with biopsy indication due to moderate hepatic steatosis in the experimental group when compared to the control group, which had a higher prevalence of biopsies due to liver disease to be elucidated (*p* = 0.019) (Table 1).

**Table 1:** Clinical profile of patients submitted to liver biopsy through the laparoscopic method (control group) and through the mini-laparoscopy method (experimental group)

		**Group**		
	**Total**	**Control**	**Experimental**	***p*-value**
**Gender**				
Female	14 (77.8%)	6 (60.0%)	8 (100.0%)	0.092
Male	4 (22.2%)	4 (40.0%)	0 (0.0%)	
**Age**				
Up to 45	8 (44.4%)	4 (40.0%)	4 (50.0%)	1.000
> 45	10 (55.6%)	6 (60.0%)	4 (50.0%)	
**Surgery**				
Video cholecystectomy	11 (61.1%)	7 (70.0%)	4 (50.0%)	0.104
Video gastroplasty	4 (22.2%)	0 (0.0%)	4 (50.0%)	
Video splenectomy	1 (5.6%)	1 (10.0%)	0 (0.0%)	
Video appendectomy	1 (5.6%)	1 (10.0%)	0 (0.0%)	
Diagnostic laparoscopy	1 (5.6%)	1 (10.0%)	0 (0.0%)	
**Reason for liver biopsy**				
Moderate hepatic steatosis	4 (22.2%)	0 (0.0%)	4 (50.0%)*	***0.019***
Severe hepatic steatosis	8 (44.4%)	4 (40.0%)	4 (50.0%)	
Hepatic cirrhosis	1 (5.6%)	1 (10.0%)	0 (0.0%)	
Liver disease to be elucidated	5 (27.8%)	5 (50.0%)*	0 (0.0%)	

^*^p < 0.05, Fisher's exact test or Pearson's chi-square (n, %)

Only one patient had an INR between 1.3–1.7, ascites or portal hypertension (*n* = 1.5.6%). Obesity was observed in four patients (*n* = 4, 22.2%) and liver with blunt edges in 14 (*n* = 14, 77.8%). The macroscopic evaluation of intraoperative steatosis in most patients was severe (*n* = 9, 50.0%), and no bile leak was observed (*n* = 0.0%); one patient in the control group developed controllable intraoperative hemorrhage (*n* = 1, 5.6%). None of these characteristics differed significantly between the groups and the quality of the biopsy sample was adequate in all samples from both groups (100.0%) (Table 2).

**Table 2:** Pre- and trans-operative profile of patients submitted to liver biopsy through the laparoscopic method (control group) and the mini-laparoscopy method (experimental group)

		**Group**		
	**Total**	**Control**	**Experimental**	***p*-value**
**INR**				
0.8–1.2	17 (94.4%)	9 (90.0%)	8 (100.0%)	1.000
1.3–1.7	1 (5.6%)	1 (10.0%)	0 (0.0%)	
**Ascites**				
No	17 (94.4%)	9 (90.0%)	8 (100.0%)	1.000
Yes	1 (5.6%)	1 (10.0%)	0 (0.0%)	
**Obesity**				
No	14 (77.8%)	9 (90.0%)	5 (62.5%)	0.275
Yes	4 (22.2%)	1 (10.0%)	3 (37.5%)	
**Portal hypertension**				
No	17 (94.4%)	9 (90.0%)	8 (100.0%)	1.000
Yes	1 (5.6%)	1 (10.0%)	0 (0.0%)	
**Liver with blunt edges**				
No	4 (22.2%)	1 (10.0%)	3 (37.5%)	0.275
Yes	14 (77.8%)	9 (90.0%)	5 (62.5%)	
**Evaluation of intraoperative macroscopic steatosis**				
Absent	3 (16.7%)	2 (20.0%)	1 (12.5%)	0.266
Mild	1 (5.6%)	1 (10.0%)	0 (0.0%)	
Moderate	5 (27.8%)	1 (10.0%)	4 (50.0%)	
Severe	9 (50.0%)	6 (60.0%)	3 (37.5%)	
**Hemorrhage**				
No	17 (94.4%)	9 (90.0%)	8 (100.0%)	1.000
Yes	1 (5.6%)	1 (10.0%)	0 (0.0%)	
**Occurrence of bile leak**				
No	18 (100.0%)	10 (100.0%)	8 (100.0%)	1.000
Yes	0 (0.0%)	0 (0.0%)	0 (0.0%)	
**Quality of biopsied sample**				
≥ 5 portal spaces	18 (100.0%)	10 (100.0%)	8 (100.0%)	1.000
< 5 portal spaces	0 (0.0%)	0 (0.0%)	0 (0.0%)	

^*^p < 0.05, Fisher's exact test or Pearson's chi-square (n, %)

**Fig. 6:**
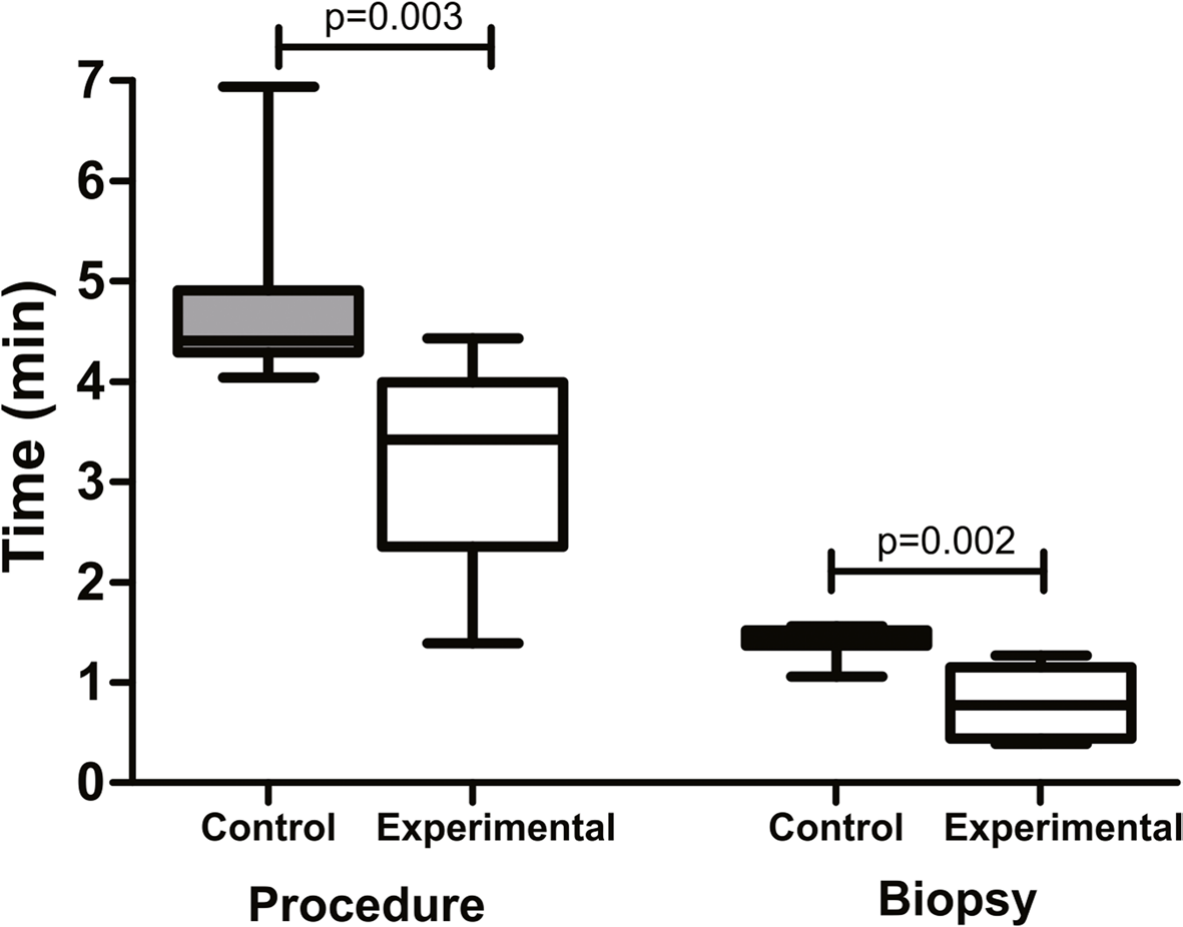
Mean time of the procedure and biopsy in patients submitted to liver biopsy through laparoscopy (control group) and mini-laparoscopy (experimental group). *p < 0.05, Mann–Whitney test (mean ± SD)

The hospital length of stay in the control group was 2.8 ± 1.7 days and 2.4 ± 0.5 days in the experimental group, with no significant difference between the groups (*p* = 0.762). The mean time of the procedure in the experimental group (3.2 ± 1.0 min) was significantly lower than that in the control group (4.7 ± 0.8 min; *p* = 0.003), as well as the time of the biopsy (0, 8 ± 0.4 min *versus* 1.4 ± 0.2 min, respectively; *p* = 0.002) (Fig. 6).

**Fig. 7:**
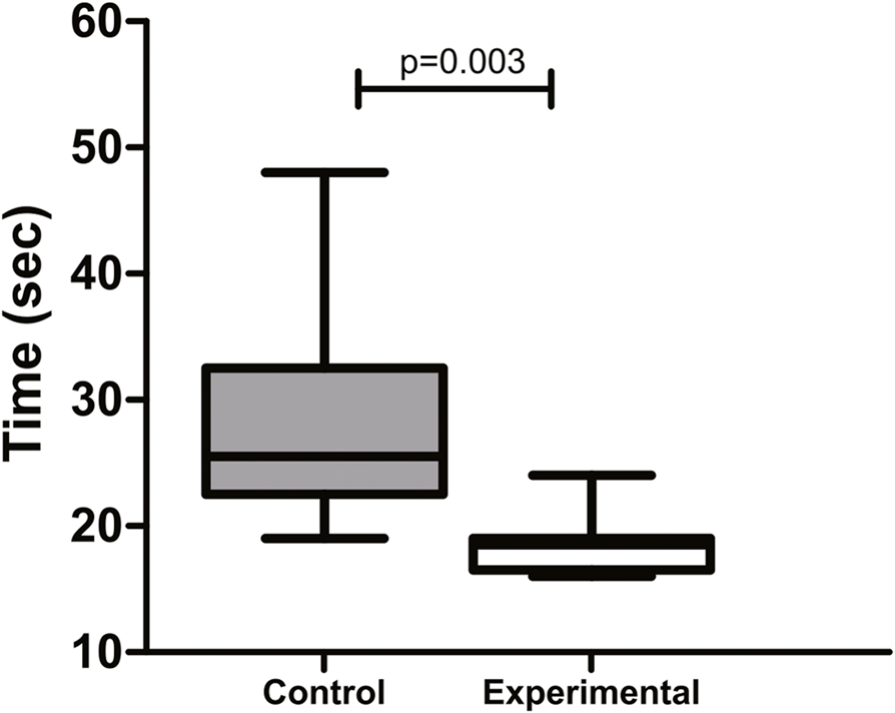
Mean time of hemostasis in patients submitted to liver biopsy through laparoscopy (control group) and mini-laparoscopy (experimental group). *p < 0.05, Mann–Whitney test (mean ± SD)

The time of hemostasis in the experimental group was also significantly shorter than in the control group (18.6 ± 2.5 s *versus* 27.9 ± 8.5 s) (*p* = 0.003; Fig. 7).

The intention-to-treat analysis showed that although the experimental group had a higher prevalence of biopsy indication due to hepatic steatosis, this factor did not interfere with the results, and the test group had a shorter mean time of procedure (*p* = 0.011), mean time of biopsy (*p* = 0.005) and time of hemostasis (*p* = 0.006) than the control group with reason for biopsy due to steatosis or cirrhosis/liver disease to be elucidated. These two subgroups did not differ significantly (Table 3).

**Table 3:** Intention-to-treat analysis of patients submitted to laparoscopic liver biopsy (control group) and mini-laparoscopy (experimental group) with different reasons for undergoing a biopsy

	**Control Group**			
	**Hepatic steatosis**	**Cirrhosis/Liver disease to be elucidated**	**Test Group**	***p*-Value**
Representative of the sample	2.00 ± 0.00	2.00 ± 0.00	2.00 ± 0.00	1.000
Hospital length of stay	2.00 ± 0.00	3.33 ± 2.16	2.38 ± 0.52	0.393
Time of procedure	5.10 ± 1.25	4.50 ± 0.40	3.17 ± 1.02*^†^	***0.011***
Time of biopsy	1.45 ± 0.09	1.39 ± 0.17	0.79 ± 0.39*^†^	***0.005***
Time of coagulation	23.50 ± 4.20	30.83 ± 9.62	18.63 ± 2.50*^†^	***0.006***

^*^p < 0.05 *versus* control group with reason for biopsy due to a diagnosis of hepatic steatosis

^†^*p* < 0.05 *versus* control group with reason for biopsy due to diagnosis of cirrhosis/liver disease to be elucidated. Kruskal–Wallis/Mann–Whitney test (mean ± SD)

The three digestive tract surgeons who proposed to use the device answered the three questions in the questionnaire at the end of the procedure. Using the Likert scale, they assigned scores from 1 to 5 that were equivalent to insufficient to excellent, respectively. All of them gave the maximum score to the eight surgical procedures in the experimental group.

There were no accidents or complications in either group, but one patient in the control group had liver hemorrhage that was promptly visualized and corrected. The presence of this complication was not statistically relevant and did not change the hospital length of stay between the groups.

## Discussion

This study showed that the device developed for video-assisted liver biopsy had a better mean procedure and biopsy time, in addition to safety and little risk of bleeding and bile leak compared to standard techniques for video-laparoscopic biopsy.

Previous studies have shown that there is no direct correlation between post-biopsy bleeding and coagulation time when the liver biopsy was performed under direct visualization by laparoscopy [17]. From then on, several guidelines emerged, which allowed liver biopsy to be performed in patients with an INR ≤ 2.0 and platelet count ≥ 25,000 µl [17, 18]. The INR was one of the parameters used to clinically define the population of this study, as all patients with an INR > 1.8 were excluded from that study.

It is important to remember that in patients who are receiving anticoagulant therapy, such as aspirin, percutaneous biopsy should only be performed at least five days after drug withdrawal [19, 20]. This can delay the diagnosis and worsen the prognosis in patients who are experiencing liver failure. In this context, laparoscopy has been considered safe in a series of cases, in patients with or without coagulopathy. However, there are still few data on the safety and usefulness of laparoscopy in patients with acute liver failure [21, 22]. Nevertheless, hemorrhage after video-assisted biopsy is often identified immediately after the procedure, which makes it safer and of immediate intervention [23].

In the present study, one patient in the control group had liver capsule hemorrhage (*n* = 1; 5.6%). It was promptly visualized and controlled, without damage to the patient's liver function. Regarding the coagulation disorder, only one patient had an INR between 1.3 and 1.7 (*n* = 1; 5.6%). None of these characteristics showed any statistical difference or led to biopsy sample damage.

As bleeding control is one of the most important factors when performing liver surgeries, the present study brings an alternative for faster and less invasive liver hemostasis. The surgical model demonstrated in the experimental group shows the use of number 14 Jelco® as a portal for the passage of the biopsy needle. The liver parenchyma fragment is biopsied, and then the needle is kept in contact with the biopsy site, and then tissue cauterization is performed by touching the monopolar electric scalpel to the needle itself (there is no transmission to the skin, subcutaneous tissue or muscle as the Jelco® polyurethane catheter is insulating). Two major advantages are obtained with this method: (1) it does not require another portal to perform the hemostasis; (2) the fact that coagulation of the parenchyma is carried out with the needle itself reduces the time to perform the procedure. These data were confirmed by the measuring the time of hemostasis, which was significantly shorter in the experimental group compared to the control group (*p* = 0.003).

Another important aspect about the liver biopsy is the size of the biopsy sample and the excessive possibility of fragmentation of the biopsied liver parenchyma into small pieces [24]. In case of unsatisfactory samples after 3 or more consecutive satisfactory biopsies attempts, it is prudent to repeat the biopsy at another time using a “Trucut” needle type [24, 25]. And as the increase in the number of punctures increases the risk of complications, it is more prudent to perform a new puncture guided by image exam or by video surgery [24, 25]. Most pathologists consider a tissue sample measuring 1.5 to 2.0 cm in length removed with a 16G needle to be satisfactory, which statistically contains at least 05 portal spaces [24–26]. The great advantage of using a “Trucut” needle is that its activation followed by the triggering already allows a 2.0 cm entry into the parenchyma, preventing fragmentated or insufficient samples. As all patients in the experimental study group were submitted to liver biopsy with a 16G Trucut needle (the same needle of choice for the percutaneous biopsy), the quality of the biopsy sample was adequate in all samples from both groups (100.0%) and there was no statistically significant difference in relation to the control group. The samples measured 2.0 cm and contained more than 05 portal spaces in both groups, which allowed the histological diagnosis of the underlying disease.

When the total time of the surgical procedure, time of biopsy and liver parenchyma coagulation time were compared, all findings were significantly lower in the experimental group than in the control group (*p* = 0.003; *p* = 0.002 and *p* = 0.003, respectively), indicating the adequate use of the device as a liver biopsy instrument, followed by satisfactory hemostasis of the biopsied area and shorter operative time.

This study has some limitations. First, the final sample size presented a lower statistical power than initially expected, recalculated with the same method at 70%. Despite not being an ideal power, this fact would contribute to not finding statistically significant differences that may exist in reality (type 2 error), so we understand that the differences found in the sample can be interpreted with confidence. In addition, laboratory-based biochemical changes were not evaluated in the present study.

## Conclusions

As it constitutes an option for liver biopsy procedure without the use of incisions, the device meets the safe and effective characteristics for minimally-invasive surgical instruments and can be used for this purpose. The greatest utility of the procedure indicated in the study is for patients who have a contraindication to perform percutaneous liver biopsy (ascites, obesity, thrombocytopenia, blood dyscrasia, portal hypertension). This reflects the potential of the device for the performance of mini-laparoscopy, and future studies may support its use in other procedures, such as renal and ovarian biopsy and in cases of peritoneal carcinomatosis.

## Data Availability

The datasets used and/or analyzed during the current study are available from the corresponding author on reasonable request.

## References

[1] Setiawan VW, Stram DO, Porcel J, Lu SC, Le Marchand L, Noureddin M (2016). Prevalence of chronic liver disease and cirrhosis by underlying cause in understudied ethnic groups: the multiethnic cohort. Hepatology.

[2] Sahara K, Paredes AZ, Tsilimigras DI, Hyer JM, Merath K, Wu L (2019). Impact of liver cirrhosis on perioperative outcomes among elderly patients undergoing hepatectomy: the effect of minimally invasive surgery. J Gastrointest Surg.

[3] Dufour DR, Lott JA, Nolte FS, Gretch DR, Koff RS, Seeff LB (2000). Diagnosis and monitoring of hepatic injury I Performance characteristics of laboratory tests. Clin Chem.

[4] Zinner M. Maingot's abdominal operations. New York City: McGraw Hill Professional; 2012.

[5] Yeo CJ (2017). Shackelford's Surgery of the Alimentary Tract.

[6] Liu A, Galoosian A, Kaswala D, Li AA, Gadiparthi C, Cholankeril G (2018). Nonalcoholic fatty liver disease: epidemiology, liver transplantation trends and outcomes, and risk of recurrent disease in the graft. J Clin Transl Hepatol.

[7] Menghini G (1958). One-second needle biopsy of the liver. Gastroenterology.

[8] Czaja AJ, Carpenter HA (2007). Optimizing diagnosis from the medical liver biopsy. Clin Gastroenterol Hepatol.

[9] Filingeri V, Sforza D, Tisone G (2015). Complications and risk factors of a large series of percutaneous liver biopsies in patients with liver transplantation or liver disease. Eur Rev Med Pharmacol Sci.

[10] Seeff LB, Everson GT, Morgan TR, Curto TM, Lee WM, Ghany MG (2010). Complication rate of percutaneous liver biopsies among persons with advanced chronic liver disease in the HALT-C trial. Clin Gastroenterol Hepatol.

[11] Rockey D, Caldwell S, Goodman Z, Nelson R, Smith A (2009). Liver AAftSo. D. Liver biopsy. Hepatology.

[12] Steele K, Schweitzer MA, Lyn-Sue J, Kantsevoy SV (2008). Flexible transgastric peritoneoscopy and liver biopsy: a feasibility study in human beings (with videos). Gastrointest Endosc.

[13] Eisenberg E, Konopniki M, Veitsman E, Kramskay R, Gaitini D, Baruch Y (2003). Prevalence and characteristics of pain induced by percutaneous liver biopsy. Anesth Analg.

[14] Phillips LD (2001). Manual de terapia intravenosa: Artmed.

[15] Nicolao C, Paczkoski RF, Ellensohn L. A história da venopunção: a evolução dos cateteres agulhados periféricos ao longo dos tempos. Rev Conhecimento Online. 2013;1:1–11.

[16] Likert  R (1961). New patterns of managemen.

[17] Kitchin D, Del Rio A, Woods M, Ludeman L, Hinshaw J (2018). Percutaneous liver biopsy and revised coagulation guidelines: a 9-year experience. Abdom Radiol (NY).

[18] O'Connor SD, Taylor AJ, Williams EC, Winter TC (2009). Coagulation concepts update. Am J Roentgenol.

[19] Burger W, Chemnitius JM, Kneissl G, Rücker G (2005). Low-dose aspirin for secondary cardiovascular prevention–cardiovascular risks after its perioperative withdrawal versus bleeding risks with its continuation–review and meta-analysis. J Intern Med.

[20] Yoon Y-I, Kim K-H, Kang S-H, Kim W-J, Shin M-H, Lee S-K (2017). Pure laparoscopic versus open right hepatectomy for hepatocellular carcinoma in patients with cirrhosis. Ann Surg.

[21] Hoffman A, Rahman F, Prengel S, Schuchmann M, Gotz M, Moehler M (2011). Mini-laparoscopy in the endoscopy unit: safety and outcomes in over one thousand patients. World J Gastrointest Endosc.

[22] Dechêne A, Sowa J-P, Schlattjan M, Wree A, Blomeyer S, Best J (2014). Mini-laparoscopy guided liver biopsy increases diagnostic accuracy in acute liver failure. Digestion.

[23] Huang JY, Lu Q, Liu JB (2018). Delayed hepatic rupture post ultrasound-guided percutaneous liver biopsy: A case report. Medicine..

[24] Filingeri V, Francioso S, Sforza D, Santopaolo F, Oddi F, Tisone G. A retrospective analysis of 1.011 percutaneous liver biopsies performed in patients with liver transplantation or liver disease: ultrasonography can reduce complications? Eur Rev Med Pharmacol Sci. 2016;20(17):3609–17. 27649662

[25] Aa B (2001). Sheth Sg Fau-Chopra S, Chopra S. Liver biopsy N Engl J Med.

[26] Scheuer PJ (2003). Liver biopsy size matters in chronic hepatitis: bigger is better. Wiley Online Library.

